# Amelioration of Cardiac Function and Activation of Anti-Inflammatory Vasoactive Peptides Expression in the Rat Myocardium by Low Level Laser Therapy

**DOI:** 10.1371/journal.pone.0101270

**Published:** 2014-07-03

**Authors:** Martha Trindade Manchini, Andrey Jorge Serra, Regiane dos Santos Feliciano, Eduardo Tadeu Santana, Ednei Luis Antônio, Paulo de Tarso Camillo de Carvalho, Jairo Montemor, Renato Oliveira Crajoinas, Adriana Castello Costa Girardi, Paulo José Ferreira Tucci, José Antônio Silva

**Affiliations:** 1 Universidade Nove de Julho, UNINOVE, São Paulo, SP, Brasil; 2 Universidade Federal de São Paulo, UNIFESP, São Paulo, SP, Brasil; 3 Heart Institute (InCor), Universidade de São Paulo, USP, São Paulo, SP, Brasil; Temple University, United States of America

## Abstract

Low-level laser therapy (LLLT) has been used as an anti-inflammatory treatment in several disease conditions, even when inflammation is a secondary consequence, such as in myocardial infarction (MI). However, the mechanism by which LLLT is able to protect the remaining myocardium remains unclear. The present study tested the hypothesis that LLLT reduces inflammation after acute MI in female rats and ameliorates cardiac function. The potential participation of the Renin-Angiotensin System (RAS) and Kallikrein-Kinin System (KKS) vasoactive peptides was also evaluated. LLLT treatment effectively reduced MI size, attenuated the systolic dysfunction after MI, and decreased the myocardial mRNA expression of interleukin-1 beta and interleukin-6 in comparison to the non-irradiated rat tissue. In addition, LLLT treatment increased protein and mRNA levels of the Mas receptor, the mRNA expression of kinin B2 receptors and the circulating levels of plasma kallikrein compared to non-treated post-MI rats. On the other hand, the kinin B1 receptor mRNA expression decreased after LLLT. No significant changes were found in the expression of vascular endothelial growth factor (VEGF) in the myocardial remote area between laser-irradiated and non-irradiated post-MI rats. Capillaries density also remained similar between these two experimental groups. The mRNA expression of the inducible nitric oxide synthase (iNOS) was increased three days after MI, however, this effect was blunted by LLLT. Moreover, endothelial NOS mRNA content increased after LLLT. Plasma nitric oxide metabolites (NOx) concentration was increased three days after MI in non-treated rats and increased even further by LLLT treatment. Our data suggest that LLLT diminishes the acute inflammation in the myocardium, reduces infarct size and attenuates left ventricle dysfunction post-MI and increases vasoactive peptides expression and nitric oxide (NO) generation.

## Introduction

The renin-angiotensin system (RAS) plays a pivotal role in the pathophysiology of myocardial infarction (MI), and in the development of heart failure [1]. In the RAS pathway, angiotensin converting enzyme (ACE) converts angiotensin I (Ang-I) into the vasoconstrictor Ang-II, which is a major effector of RAS, and is therefore responsible for most of its pathophysiological effects [2]. Although found in the systemic circulation, Ang-II is also produced in the cardiac tissue by a local RAS [3]. Ang-II has been shown to increase oxidative stress, which could in turn activate inflammatory [4] and apoptotic [5] pathways. In fact, RAS blockade with ACE inhibitors (ACEis) or angiotensin receptor blockers (ARBs) has been shown to ameliorate several pathological cardiac conditions. This molecular suppression improves the outcome of cardiac remodeling [6]–[9]. A vasoactive system, the kallikrein-kinin system (KKS), is also produced locally in the cardiac muscle. KKS is markedly affected by ACEi therapy. Diminished degradation of bradykinin (BK), a potent vasodilator kinin peptide, has been reported to occur following ACE inhibition [10], [11]. Kinins exert their pharmacological actions through two G-coupled transmembrane receptors, kinin B1 and B2 [12]. Whereas kinin B2 is expressed constitutively in most cells and tissues in mammals, kinin B1 is an inducible receptor that is expressed mostly in inflammatory states [12].

Until the discovery of the enzyme ACE2, which cleaves Ang-II to generate the vasodilator and anti-fibrotic peptide Ang1–7 [13], BK was known as the most antagonistic physiological response to RAS activation. Increased BK availability was found to be an important component of the success of ACEis [10]. ACE2 expression and activation was noted to increase after experimental MI, possibly circumventing the deleterious effects of RAS. The peptide Ang1–7 is found in the heart and kidneys, and in most tissues related to cardiovascular system homeostasis [2], [14]. Ang1–7 is implicated in the prevention of Ang-II-induced cardiovascular hypertrophy and remodeling by activation of Mas receptor [15]–[18].

Low-level laser therapy (LLLT) has become an alternative therapy to modulate various biological processes. Depending on the wavelength, dosage, and condition of the irradiated tissue, LLLT can induce an anti-inflammatory effect, reduce pain, and accelerate cell proliferation [12], [19]–[23]. We previously showed that LLLT was effective at modulating the kinin B1 and B2 receptors mRNA expression in the subplantar muscle of rat paw carrageenan-induced inflammation [12]. In addition, using the same model, we reported that both plasma and tissue pre-kallikrein mRNA expression were modulated after LLLT [22].

The use of LLLT to treat pathophysiological conditions started four decades ago [24]. To the best of our knowledge, the first report of LLLT usage on experimental MI was published in 2000 [25], and demonstrated that laser irradiation could attenuate infarct-associated remodeling. MI comprises the ischemic area of the myocardium that is subserved by an occluded coronary artery. Immediately after MI, tissue injury and death of cardiomyocytes trigger a synchronized acute inflammatory response that can last hours, days, or weeks (for review, see [26]). Several pro-inflammatory cytokines and chemokines, such as interleukin (IL)-1 beta, IL-6, IL-8, and tumor necrosis factor α (TNFα) are upregulated during this inflammatory phase [27]. Although some studies have used LLLT to treat cardiac dysfunction and reduce the myocardial infarct size after laser irradiation [28]–[30], the molecular effects of the laser on the myocardium remain controversial. To date, no study using MI rat models treated with LLLT has assessed vasoactive peptide expression. As inflammation appears secondary to myocardial hypoxia due to the MI, it is possible that LLLT could reduce cytokine expression [19]. We therefore hypothesized that LLLT diminishes the acute inflammatory response in the myocardium after MI and ameliorates cardiac function by upregulating the expression of vasoactive peptides and potentiating the nitric oxide (NO)-mediated vasodilation. The generation of short-lived uncharged free radical NO might prevent the decrease in oxygen and nutrients caused by obstruction of the tissue's blood supply. To this end, we examined the effect of LLLT on the expression of KKS and RAS components in rats with MI, the plasmatic nitric oxide metabolites concentration and myocardial capillaries density. The effect of LLLT on left ventricular (LV) geometry and function was also assessed.

## Materials and Methods

### Ethics statement

All the experimental procedures were performed to the *Guide for the Care and Use of Laboratory Animals* published by the US National Institutes of Health (NIH publication no. 85–23, revised 1996). The project research was approved by the Institutional Research Ethics Committee of the Nove de Julho University (Number 0015/2012), São Paulo, Brazil. All surgery was performed under ketamine and xylazin anesthesia, and all efforts were made to minimize suffering.

### Animals and MI surgical procedure

Female Wistar rats (n = 82) with 10 weeks of age were subjected to thoracotomy and infarction by coronary occlusion. The surviving rats were randomly divided into infarcted non-treated group (MI, n = 28) and infarcted laser-treated group (MI+Laser, n = 30). Rats that received the same surgical procedure for thoracotomy without coronary ligation served as control group (Control, n = 14). For MI induction, the descending left coronary artery was occluded near its origin under ketamine (50 mg/kg) and xylazin (10 mg/kg) anesthesia as previously described [31]. All parameters evaluated in this study were analyzed 3 days after MI.

### Low level laser therapy

After surgery, the animals were immediately randomized into two experimental groups (with or without LLLT). The laser device used was an Aluminum Indium Gallium Phosphorus – AlGaInP (Twin Laser – MM Optics, São Carlos, SP, Brazil) with wavelength 660 nm, power 15 mW, laser beam spot size 0.785 cm^2^, energy density 22.5 J/cm^2^, irradiation time 60 sec, and energy delivered 1.1 J. The laser dose used in this study was similar to laser parameters previously described [28]. The laser wavelength was chosen accordingly beneficial effects of LLLT previously reported [32]. The laser beam was placed in contact with the myocardium surface corresponding to the infarcted area. After ligation as describe above, the heart was put in the chest to recover itself for 60 sec and then the heart was put out and random to receive or not the laser irradiation. The optical fiber was fixed with a delivery arm and precisely positioned with the fiber tip 3 cm above the myocardium. This allowed for a laser beam spot size of 0.785 cm^2^.

### Assessment of MI size, geometry and function of LV

Three days after descending left coronary artery occlusion or sham surgical procedure, animals were anesthetized as described above (K-X mixture) and LV measurements were performed using a 12-MHz transducer connected to a HP Sonos-5500 echocardiograph (Hewlett–Packard, Palo Alto, CA, USA). The MI size was evaluated on transversal 2-dimensional view of the LV and expressed as the proportion of the LV perimeter as previously described [31], [33]–[35]. The MI was defined by echocardiography as any segment with increased echogenicity and/or change in myocardial thickening or systolic movement (hypokinesia, akinesia, or dyskinesia). The diastolic (DA) and systolic (SA) LV areas were measured by 2-dimensional images at the basal, midview, and apical view. Diastolic function was evaluated based on the parameters of mitral inflow and LV outflow tract velocity curves by pulsed wave Doppler and with tissue Doppler imaging of mitral lateral annulus. From the mitral diastolic flow velocity curve, the maximum velocity of the E and A waves were measured and the E/A ratio calculated. Isovolumic relaxation time was obtained by measuring the interval between the end of the systolic spectral curve of the LV outflow tract and the beginning of the mitral diastolic spectral curve (E wave). E-wave deceleration time was measured, as the interval from the peak of the E wave to the point the deceleration ramp would reach the baseline.

### Biometric data and biological sample

After echocardiographic analyses, rats received an overdose of urethane (4.8 g kg^−1^, i.p.), the right carotid artery was cannulated and arterial blood was quickly withdrawn for determination of plasma kallikrein concentration and nitric oxide metabolites. Plasma was isolated from blood and frozen until assay. The heart and left lung were immediately removed. The LV weight was used as an indicator of myocardial hypertrophy. The lung wet weight (WW) and dry weight (DW) were measured before and after tissue samples were dried at 70°C, respectively, to determine lung water content (H_2_O): H_2_O (%) = [(WW−DW)/WW]×100. LV fragments of remote area to MI were placed in 5% saline solution to remove excess blood. The myocardial tissue was stored in cryogenic tube and kept frozen in liquid nitrogen for molecular analysis. Quantitative PCR was performed on all LV remote area samples from the 3 experimental groups. Western blot analysis was performed on pool samples of 5 animals per group.

### Gene expression quantification

Total RNA was extracted from left ventricle (LV) remote area samples and Real-time PCR assay was performed to access mRNA quantification. Thawed tissues were homogenized in 1 ml of TRIzol reagent (Gibco BRL, Gaithersburg, MD) and total RNA was isolated accordingly to the manufacturer's instructions.

One microgram of total RNA was used for cDNA synthesis and Real-Time PCR gene expression analysis. Initially, contaminating DNA was removed using DNase I (Invitrogen) at a concentration of 1 unit/µg RNA in the presence of 20 mM Tris-HCl, pH 8.4, containing 2 mM MgCl_2_ for 15 min at 37°C, followed by incubation at 95°C for 5 min for enzyme inactivation. Then, the reverse transcription (RT) was carried out in a 200 µl reaction in the presence of 50 mM Tris-HCl, pH 8.3, 3 mM MgCl_2_, 10 mM dithiothreitol, 0.5 mM dNTPs, and 50 ng of random primers with 200 units of Moloney murine leukemia virus-reverse transcriptase (Invitrogen). The reactions conditions were: 20°C for 10 min, 42°C for 45 min and 95°C for 5 min. The reaction product was amplified by real time PCR on the 7500 Sequence Detection System (ABI Prism, Applied Biosystems, Foster City, CA) using the SYBR Green core reaction kit (Applied Biosystems). The thermal cycling conditions were: 50°C for 2 min, then 95°C for 10 min, followed by 40 cycles at 95°C for 15 s and 60°C for 1 min. Experiments were performed in triplicates for each data point. Target gene mRNA abundance was quantified as a relative value compared with an internal reference, GAPDH, whose abundance was believed not to change between the varying experimental conditions. Primers sequences used for quantitative PCR mRNA quantification for kinin B1 and B2 receptors, Mas receptor, interleukins 1-beta and 6, ACE, ACE2, iNOS, eNOS and GAPDH are listed in Table 1. One microliter of RT reaction was used for Real-Time PCR.

**Table 1:** Rat primers used for Real-time PCR mRNA quantification.

Gene	Forward Primer	Reverse Primer	Accession Number GenBank
Kinin B1 receptor	5′-CCTTCCAGGCTT	5′GGTTGGAGGATT	NM_030851.1
	AAACGATTCTC-3′	GGAGCTCTAGA-3′	
Kinin B2 receptor	5′-CCACCACGGCCT	5′-CGAACAGCACC	NM_001270713.1
	CTTTCAG-3′	CAGAGGAA-3′	
Interleukin 6	5′-GAGGAGACTTCA	5′- TCCTTAGCCACT	NM_012589.2
	CAGAGGAT-3′	CCTTCTGT-3′	
Interleukin1beta	5′-CAGGAAGGCAGT	5′- GGGATTTTGTC	M98820.1
	GTCACTCA-3′	GTTGCTTGT-3′	
ACE	5′-CACCGGCAAGGT	5′- CTTGGCATAGTT	NM_012544.1
	CTGCTT-3′	TCGTGAGGAA-3′	
ACE2	5′- GCCAGGAGATG	5′- CTGAAGTCTCC	NM_001012006.1
	ACCGGAAA-3′	ATGTCCCAGATC-3′	
Mas receptor	5′- CATCTCTCCTCT	5′- CCTCATCCGGA	NM_012757.2
	CGGCTTTGTG-3′	AGCAAAGG-3′	
iNOS	5′-GATCAATAACCT	5′-GCCCTTTTTTGC	NM_012611.3
	GAAGCCCG-3′	TCCATAGG-3′	
eNOS	5′-CCGCACTTCTGT	5′-GCTCGGGTGGAT	NM_021838.2
	GCCTTTGCTC-3′	TTGCTGCTCT-3′	
GAPDH	5′-TGCACCACCAAC	5′-GCCCCACGGCC	NM_017008
	TGCTTAGC-3′	ATCA-3′	

Quantitative values for target gene and GAPDH mRNA transcription were obtained from the threshold cycle number, where the increase in the signal associated with an exponential growth of PCR products begins to be detected. Melting curves were generated at the end of every run to ensure product uniformity. The relative target gene expression level was normalized on the basis of GAPDH expression as an endogenous RNA control. ΔCt values of the samples were determined by subtracting the average *C_t_* value of target gene mRNA from the average *C_t_* value of the internal control GAPDH. The 2^−ΔΔCt^ parameter was used to express the relative expression data.

### Western blot analysis

LV remote area protein homogenates were obtained by homogenizing the tissues in a Polymix PX-SR 50 E homogenizer (Kinematica AG, Switzerland) in an ice cold buffer containing PBS (10 mM phosphate, 140 mM NaCl, pH 7.4), protease inhibitors (1 mM pepstatin, 1 mM leupeptin, and 230 mM PMSF) and phosphatase inhibitors (15 mM NaF and 50 mM sodium pyrophosphate) plus 1∶300 Phosphatase Inhibitor Cocktail 2 (Sigma-Aldrich, St. Louis, MO, USA). Protein concentration was determined by the Lowry method [36].

Homogenate protein samples (30 µg) and 0.75 µl of plasma were subjected to sodium dodecyl sulfate polyacrylamide gel electrophoresis (SDS-PAGE) in 10% polyacrylamide gels. Separated proteins were transferred onto hydrophobic polyvinylidene difluoride (PVDF) membranes (Hybond-P, Amersham Biosciences; Piscataway, J, USA), and the transfer efficiency was monitored with 0.5% Ponceau S staining of the blot membrane. The PVDF membranes were soaked in a blocking buffer (5% nonfat dry milk and 0.1% Tween 20 in PBS, pH7.5) for 1 h at room temperature and then incubated overnight at 4°C with primary antibody at the following concentrations: goat anti mas receptor antibody (1∶200 dilution; Santa Cruz Biotechnology, Santa Cruz, CA, USA), goat anti-VEGF (1∶1,000; Abcam, Cambridge, MA, USA), goat anti- plasma kallikrein (1∶1,000; Santa Cruz Biotechology, Santa Cruz, CA, USA), anti-GAPDH (1∶500; Santa Cruz Biotechnology, Santa Cruz, CA, USA), anti-beta actin (1∶50,000; Merck, Darstadt, Germany) and anti-albumin (1∶500; Santa Cruz Biotechnology, Santa Cruz, CA, USA). After overnight incubation, membranes were washed five times and then incubated for 1 h with horseradish peroxidase-conjugated goat anti-mouse, goat anti-rabbit and rabbit anti-goat secondary antibodies (1∶2,000; Invitrogen, San Diego, CA, USA). Membranes were again washed five times with blocking buffer and then rinsed twice in PBS. Bound antibody was detected using an enhanced chemiluminescence reagent for 1 min. The bands were visualized and digitized using the ImageScanner LAS4000 mini (GE HealthCare, Little Chalfont, UK) and quantified using ImageJ software (Bethesda, MD, USA). Identical amounts of protein were loaded into each well of the gel, GAPDH, beta-actin or albumin expression levels were used as loading controls and to normalize the data.

### Histology and periodic acid-Schiff (PAS) staining

The hearts were removed three days after infarction or sham surgery and fixed in 4% buffered formaldehyde overnight. The LV fragments were washed with PBS, dehydrated through a graded series of ethanol, diaphonized with Xylol and embedded with paraplast. Thereafter, the samples were cut into sections of 3 µm thick and stained with periodic acid-Schiff (PAS) for capillary density quantification. Capillary densities were evaluated in 6 randomized 400×magnication using a Nikon Eclipse E200 microscope and Nikon Infinity Optical System (Kurobane Nikon Co., Tochigi, Japan), and the software Image Pro-Plus 4.0 (Media Cybernetics Inc., Rockville, MD, USA). Capillarity was expressed as the total amount per area (capillaries/mm^2^).

### Plasma nitric oxide metabolites (NOx) measurement

Nitrite/nitrate concentration (NO_x_), an indirect measure of NO levels, was determined as previously described [37]. Blood cells were removed by centrifugation (10 minutes at room temperature at 1.200×g). Plasma was filtered through a 10 kDa filter (Centricon 10, Millipore, Billerica, MA, USA) for 1 hour (4°C at 10.000 rpm). Samples of the filtrate were maintained at −20°C until the day of the measurements. For plasma determination of NO_x_, nitrate was converted to nitrite by nitrate reductase (*Aspergillus niger*, Sigma-Aldrich, St. Louis, MO, USA) and all measurements were performed in duplicate. Samples (10 µL) were incubated with 1 µM NADPH to initiate the reaction plus 14 mU of the enzyme. The final volume was adjusted with a phosphate buffer (to 100 µL). After 1-hour incubation at room temperature, 10 µL of freshly prepared solution of DAN (2,3-diaminonaftalen 0.05 mg/ml in 0.62 M HCl) was added and samples were immediately vortexed. DAN reacts with nitrite in the acidic assay medium to generate 1-(H)-naftotriazole, a fluorescent product. After 10 minutes incubation, the reaction was terminated by adding 10 µL of NaOH (2.8 N). Usually a conversion greater than 90% was obtained within the first minute of mixing. The concentration of nitrite in the samples was obtained by measuring the fluorescence of 1-(H)-naftotriazole by excitation at 365 nm and emission at 450 nm. The final concentration was calculated by using a standard curve created by subtracting the calculated enzyme “blank” (nitrate reductase plus NADPH) values from the values obtained in the experiment.

### Statistical analysis

Data were analyzed with GraphPad Prism software (La Jolla, CA, USA). The Shapiro-Wilk and Levene tests were used to verify normality and error variances, respectively. Results were evaluated using Student's *t*-test for comparisons of MI size for two groups and with Chi-squared test for analysis of mortality and frequency. One-way ANOVA complemented by Newman–Keuls test was used to detect differences between three groups at sample with normal distribution. However, Kruskal-Wallis followed by Dunn's multiple comparison tests was applied for no-normality data. A p value ≤0.05 was considered significant with two-tailed probability and the results are expressed as mean ± standard error of the mean (SEM).

## Results

### LLLT ameliorated cardiac function and reduced infarction size

A total of 82 rats were subjected to MI surgical induction; 12 of them died immediately after coronary occlusion. Thus, the infarcted group comprised 38 and 32 rats with (MI+Laser) and without (MI) laser therapy, respectively. One animal from the treated group was excluded because it did not present with MI. A small number of animals died three days post-MI, 4/32 and 7/37 in the MI+Laser and MI groups, respectively, with no statistical difference (p>0.05, Chi-squared test).

**Figure 1:**
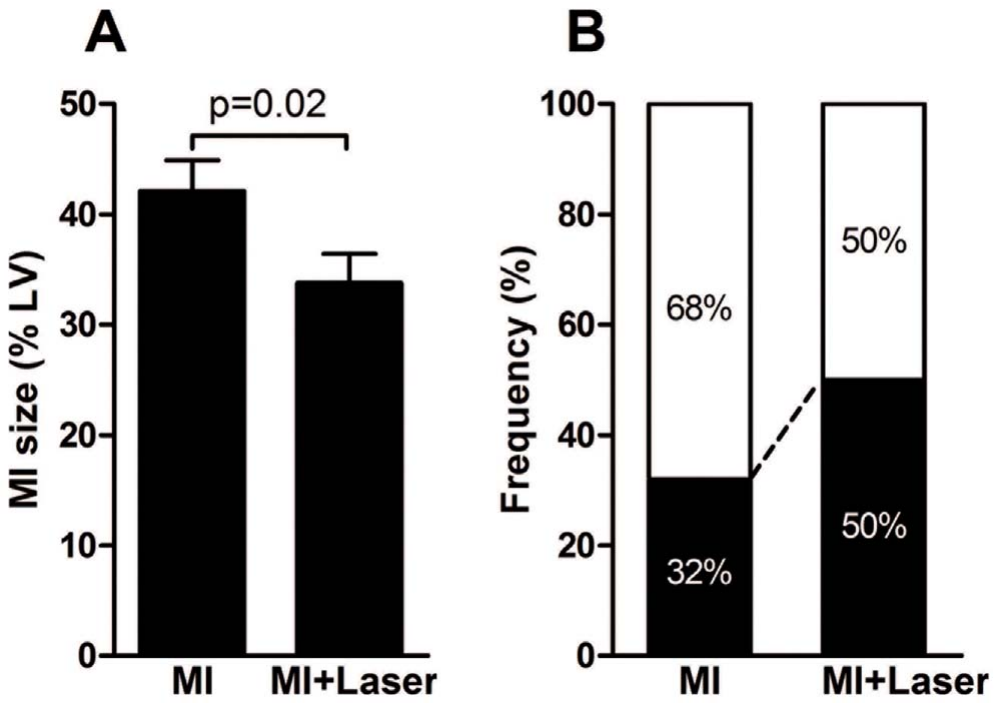
Myocardial infarction size after LLLT. (**A**) Size of the infarct as a percentage of left ventricular (LV) perimeter in infarcted rats (MI) and infarcted rats treated with laser (MI+Laser) after three days of descending left coronary artery occlusion. (**B**) Frequency of large (□) or small plus mild (▪) infarctions (p value = 0.004, Chi-squared test).

Figure 1A shows the mean infarction size in the MI and MI+Laser groups. The size of infarction was notably smaller in the MI+Laser group compared with the MI group (p = 0.02, Student's *t*-test). The size of infarction was categorized according to a cut-off point of 37% LV (Figure 1B) as previously described [33]. Importantly, the number of large infarcts (>40%) was significantly lower in the laser-treated group compared with the non-treated group (p = 0.004, Chi-squared test).

Table 2 shows the biometric and echocardiographic data. The body weight values were similar among all groups, and the MI did not induce changes in myocardial mass. Although the mean value of pulmonary water content was increased in the MI group, no significant difference among the three experimental groups was found.

**Table 2:** Biometric and echocardiographic data 72 hours after coronary occlusion and laser therapy.

	Experimental groups			
Variables	Control (n = 14)	MI (n = 28)	MI+Laser (n = 30)	*p* value
*Biometric*				
BW (g)	206±6	200±4	201±5	= 0.7
LV (mg)	681±39	693±47	751±28	= 0.3
LV/BW (mg/g)	3.6±0.2	3.0±0.4	3.8±0.2	= 0.5
H_2_O (%)	79.0±0.3	81.0±0.5	79.0±0.8	= 0.06
*Echocardiography*				
LVDA (mm^2^/BW)	0.01427±0.00070	0.01611±0.00110	0.01593±0.00070	= 0.3
LVSA (mm^2^/BW)	0.0038±0.0001*	0.0116±0.0008	0.0103±0.0003	<0.0001
FAC (%)	72±1*	27±2	35±1^#^	<0.0001
E Wave (cm/s)	0.69±0.03	0.68±0.05	0.73±0.03	= 0.4
A Wave (cm/s)	0.28±0.01	0.32±0.07	0.25±0.08	= 0.055
E/A ratio	2.6±0.2∧	4.5±0.9	5.0±0.7	= 0.02

BW, body weight; FAC, fractional area change; LV, left ventricle; H_2_O, pulmonary water content; LVDA, left ventricular diastolic area; LVSA, left ventricular systolic area; E, E wave; A, A wave; E/A ratio, relation between velocity of E and A waves. One-way ANOVA was applied in comparisons. Values are means ± SEM. *p<0.0001 *vs*. MI and MI+Laser; ^#^ p<0.0001 *vs*. MI; ∧p = 0.02 vs. MI and MI+Laser.

Regarding the transthoracic echocardiography, MI induced a significant increase in LV systolic area; however, LLLT did not affect the LV alterations induced by MI. The MI resulted in a remarkable reduction in the fractional area change (FAC) in laser-treated and non-treated rats compared with the Control group. Interestingly, our data suggests that LLLT attenuated the systolic dysfunction provoked by MI. Although E and A waves were not altered post-MI on pulsed Doppler, rats presented a restrictive LV filling pattern defined as an increased ratio of early (E) to late (A) filling velocities and rapid deceleration of the early filling wave. LLLT did not change these parameters.

### Anti-inflammatory response induced by LLLT

**Figure 2:**
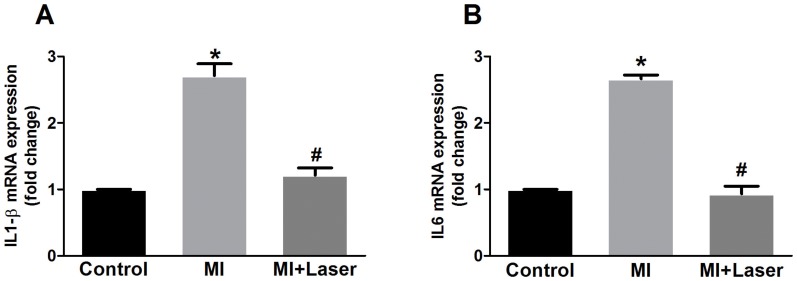
mRNA expression of interleukins 1 beta (IL-1beta) and 6 (IL-6) on myocardial tissue after LLLT. Myocardial infarction remote area presented an acute inflammation of the myocardium, as observed as an increased of cytokines IL-1beta and IL-6 mRNA in the left ventricles (LV) of MI rats (n = 28). A downregulation of IL-1beta (**A**) and IL-6 (**B**) mRNA expression was detected in the group MI+Laser (n = 30), suggesting an anti-inflammatory effect of LLLT. Control group was composed of 14 rats. Data are means ± S.E.M. *p<0.05 *vs.* Control group; ^#^p<0.05 *vs.* MI group. P values given were determined by ANOVA.

**Figure 3:**
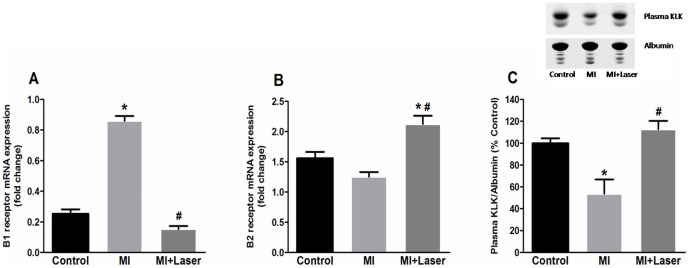
Effects of LLLT on kinins receptors mRNA expression on myocardial tissue and circulating levels of plasma kallikrein. Distinct kinin receptors mRNA modulation was observed in the remote area of MI. An increased kinin B2 receptor mRNA expression (A) and a diminished kinin B1 mRNA expression (B) was observed in the left ventricles (LV) of MI+Laser rats (n = 30). Control and MI groups were composed of 14 and 28 rats, respectively. Increased circulating levels of plasma kallikrein (C) were detected after LLLT. Data are means ± S.E.M. *p<0.05 *vs.* Control group; ^#^p<0.05 *vs.* MI group. P values given were determined by ANOVA.

Gene expression of IL-1beta and IL-6 strongly increased 3 days after MI. LLLT after coronary occlusion significantly reduced the mRNA expression of these cytokines to values that were similar to those of control rats (Figure 2A and 2B). MI resulted in distinct kinin receptor mRNA expressions. Whereas the kinin B1 mRNA content was considerably increased after MI, kinin B2 mRNA expression did not change 3 days after MI. Kinin B2 receptor mRNA expression was distinctly augmented after MI and LLLT, whereas LLLT significantly decreased the kinin B1 receptor mRNA content after MI (Figure 3A and 3B). The circulating levels of the bradykinin-forming enzyme plasma kallikrein were also evaluated. As seen if Figure 3C, the levels of plasma kallikrein significantly increased after LLLT as compared to non-treated post-MI rats.

**Figure 4:**
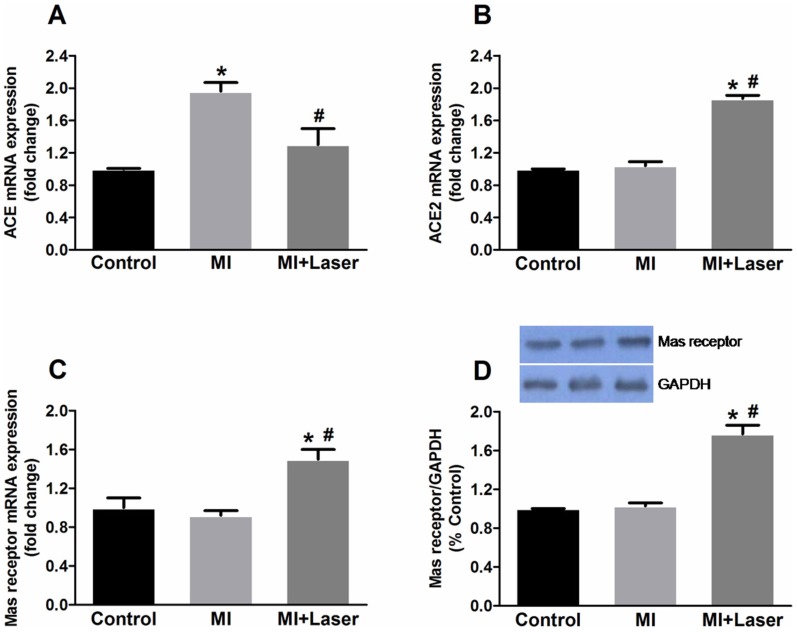
Quantification of ACE and ACE2 mRNA, and Mas receptor mRNA and protein on myocardial tissue. An up-regulation of ACE mRNA (**A**) was detected after MI. This effect was blunted by LLLT. ACE2 mRNA (**B**) was strongly expressed in in the LV of MI+Laser (n = 30). Control and MI groups were composed of 14 and 28 rats, respectively. An up-regulation of Mas gene (**C**) and protein (**D**) expression was detected in MI+Laser rats. Data are means ± S.E.M. *p<0.05 *vs.* Control group; ^#^p<0.05 *vs.* MI group. P values given were determined by ANOVA.

ACE and ACE2 mRNA contents were also quantified (Figure 4A and 4B, respectively). MI markedly increased ACE mRNA expression 3 days after coronary occlusion. LLLT significantly reduced ACE mRNA expression in the myocardium after MI (Figure 4A). MI did not change ACE2 mRNA expression; however, in the MI+Laser group, ACE2 gene expression was augmented when compared to all experimental groups (Figure 4B). Similarly, the gene expression of the Mas receptor significantly increased in the MI+Laser group (Figure 4C). In addiction, Mas receptor protein expression also increased in the infarcted myocardium after LLLT (Figure 4D).

### Increased generation of NO metabolites by LLLT

**Figure 5:**
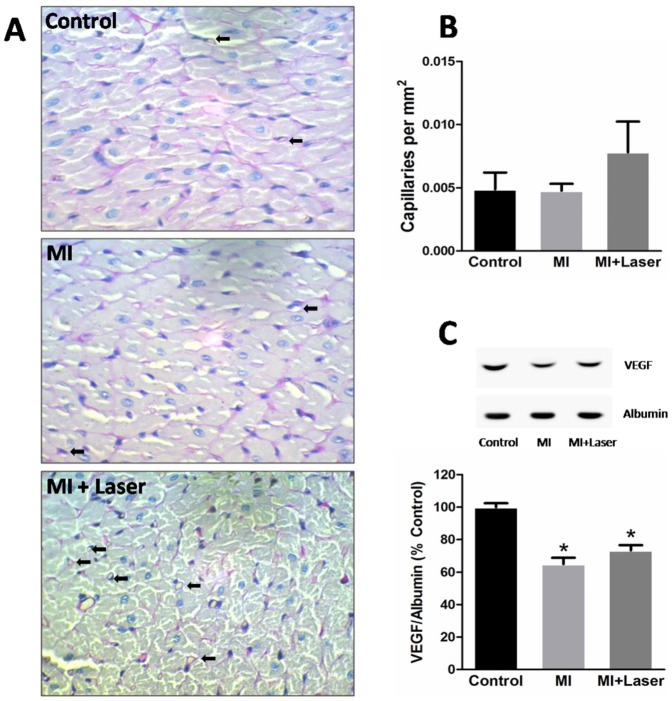
Histological changes and evaluation of capillaries density and VEGF levels of the remote myocardium after LLLT. Representative microphotographs of the remote myocardium stained with (**A**) Periodic acid-Schiff for capillary counting (400× original magnification, arrows indicate some capillaries). Parameters including capillarity (**B**), and myocardial VEGF levels (**C**) were evaluated in remote myocardial samples from experimental groups. Control, MI and MI+Laser groups were composed of 7, 12 and 14 rats, respectively for each evaluation. Data are means ± SEM. *p<0.05 *vs.* Control group. P values given were determined by ANOVA.

Angiogenesis post-MI was also evaluated in the myocardial remote area after LLLT. Periodic acid Schiff (PAS) staining was used to count capillaries density. As depicted in Figures 5A and 5B, we were unable to find evidences that LLLT could increase neovascularity. In addition, VEGF protein expression did not reach significant difference between laser-irradiated and non-irradiated myocardium (Figure 5C).

**Figure 6:**
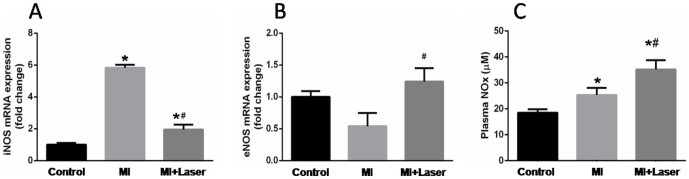
Analyses of iNOS and eNOS mRNA content on myocardial tissue and serum total nitrites concentration. A diminished iNOS mRNA expression (**A**) was observed after LLLT. Increased myocardial eNOS mRNA content (**B**) and plasma NO metabolites (**C**) were found after LLLT. Control, MI and MI+Laser groups were composed of 14, 28 and 30 rats, respectively. Data are means ± SEM. *p<0.05 *vs.* Control group; ^#^p<0.05 *vs.* MI group. P values given were determined by ANOVA.

The iNOS mRNA expression increased 3 days after MI, however, we observed a diminished iNOS gene expression after LLLT (Figure 6A). Interestingly, endothelial NO synthase was significantly augmented in the remote myocardium after LLLT (Figure 6B). Moreover, plasma nitrite and nitrate concentrations (NOx) were markedly high with LLLT (Figure 6C).

## Discussion

The major findings of the present study can be summarized as follows: (i) Low level laser therapy applied immediately after MI significantly improves cardiac function. (ii) LLLT decreases pro-inflammatory cytokines mRNA expression. (iii) LLLT post-MI augments LV mRNA expressions of kinin B2 receptor and ACE2, and Mas receptor mRNA and protein content. In addition, the circulating levels of plasma kallikrein markedly increase after laser irradiation. (iv) LLLT fails to increase either capillaries density or VEGF protein content in the remote area of MI. (v) Finally, whereas a diminished iNOS mRNA content is observed after LLLT, eNOS mRNA content and plasma NOx concentration are remarkably increased after LLLT, suggesting that NO generation may participate of the cardioprotective mechanism elicited by LLLT.

Increased kinin B1 receptor expression after inflammatory insult has been observed in independent studies [38], [39]. We previously showed a diminished kinin B1 receptor expression after LLLT in the inflamed subplantar muscle [12]. Kinin B1 is expressed as an inducible receptor [39]–[43] whose expression depends on the strength of the inflammatory stimulus. As expected, MI-related inflammation augmented B1 receptor mRNA expression. Interestingly, this event was blunted by LLLT. This observation, taken together with the reduced mRNA content of IL-1beta and IL-6 in the MI+Laser group, suggests that the LLLT is effective to downregulating inflammatory mediators mRNA expression after MI as previously described [44].

In the current study, we demonstrate that LLLT was capable of increasing kinin B2 receptor mRNA expression in the myocardium. As kinins are continuously released during cardiac hypoxia and ischemia [45], [46], the binding of kinins to endothelial B2 receptors may lead to the release of nitric oxide (NO) and prostacyclin (PGI2), exert a vasodilator effect, and preserve myocardial stores of energy-rich phosphates and glycogen [38], thus eliciting cardioprotection [40]. Therefore, upregulation of kinin B2 receptor mRNA expression in cardiac muscle after LLLT may improve tissue recovery after MI by modulating vasodilation. Our observation of an increased eNOS mRNA expression in the myocardium and subsequently, an augmented plasma NOx concentration after LLLT implies that the short-lived uncharged free radical NO could be involved in the cardioprotection elicited by LLLT. A feasible NO-mediated vasodilation might lead to a better blood irrigation in the border zone and the remote areas of the infarction. In fact, a study reported an augmented eNOS expression after LLLT using He–Ne laser at 632.5 nm to stimulate human umbilical vein endothelial cell (HUVEC) [47]. eNOS produces NO constitutively at low levels but eNOS activity can be transiently stimulated by hormones or environmental stimuli [47]. Our findings indicated that the increased plasma NO metabolites upon LLLT was consistent with the ability to up-regulate eNOS gene.

Furthermore, stimulation of kinin B2 or B1 receptors differentially activates eNOS or iNOS, respectively [48]. In healthy endothelium, the kinin B2 receptor activates eNOS resulting in a short burst of Ca^2+^-dependent NO production [49]. Under inflammatory conditions, endothelial kinin B1 receptor stimulation leads to acute activation of iNOS via ERK1/2-dependent phosphorylation at Ser^745^ and prolonged and high output NO production [50], [51]. Our results indicate that LLLT increases eNOS mRNA expression to the basal levels after MI. On the other hand, kinin B1 receptor mRNA expression was lower after LLLT in irradiated myocardium. This latter data corroborates the diminished iNOS mRNA expression observed after LLLT.

The current study brings new insight into the effects of LLLT on plasma serine proteases levels. In fact, the augmented circulating levels of plasma kallikrein found in our experimental model evidences the role of kinin in mediating the cardioprotective actions of LLLT. In this context, plasma kallikrein and bradykinin augmentation have been shown to be associated with the cardioprotective effects of exercise in hypertensive subjects [52]. On the contrary, reduced KKS activity in the myocardium facilitates development of cardiac failure [38], [53]. In fact, reduced titers of plasma kallikrein, high molecular weight kininogen, and plasma thromboplastin antecedent (factor XI) were observed in the plasma samples of patients with acute myocardial infarction in comparison to control samples [54].

The results obtained in this study also highlight the RAS participation in the anti-inflammatory LLLT response. The observation that ACE mRNA content was increased 3 days after MI corroborates the results of several previous studies of experimental MI and of humans with MI and cardiac failure [55]. We also demonstrate for the first time that LLLT significantly diminished ACE mRNA expression after MI, although not to the levels found in control animals.

Several studies have reported that ACE2 expression is a protective counterbalance of RAS [56], [57]. The increase of ACE2 content after MI has been reported in humans and rodents. Patients with ischemic heart failure showed increased ACE and ACE2 immunoreactivity in the myocardium when compared to healthy subjects [57]. MI in Sprague-Dawley rats resulted in increased cardiac ACE and ACE2 mRNA compared to control rats [57]. Our study indicates that the ACE2 mRNA augmentation observed after MI and followed by LLLT may contribute to cardioprotection.

Of particular note is that the augmentation of ACE2 expression was coordinated with an increase in Mas receptor expression. This synchronized expression of both ACE2 and Mas receptor has been observed in various studies [58]–[59]. These observations suggest that Ang1–7 might regulate the effects of LLLT on the myocardium, since the Mas receptor is activated exclusively by Ang1–7 [60]. In fact, Ang1–7 is known to exert several cardioprotective actions in the heart (for review, see [2]). *Mas* receptor gene ablation abolishes the binding and renal activity of Ang1–7 in mice. Mas receptor overexpression increases Ang1–7 activity, which can be blocked by the specific Ang1–7 antagonist, A-779 [61]. Some authors have reported that loss of Ang1–7 immunoreactivity after MI within the infarcted area contrasts with an apparent increased expression of the peptide in the zones bordering the infarcted region of the LV [62]. Intravenous infusion of Ang1–7 significantly diminished the left ventricular end-diastolic pressure, and preserved coronary flow and endothelial function [63]. Moreover, *mas* knockout mice showed an impaired cardiac function and structure [64]. Interestingly, Ang 1–7 may also enhance NO generation, especially through Mas receptor activation [65]. This observation, and the increased Mas gene and protein expression observed in our study, corroborate to the hypothesis that vasoactive peptides receptors activation may lead to NO formation after LLLT.

From the above results, a picture is emerging in favor of the participation of the ACE2-Ang1-7-Mas receptor axis and kinins in the protective action of LLLT. Taken together, the improved cardiac function data we presented here might be at least partially related to the increase in vasoactive peptides expression in the MI+Laser group. For instance, another study reported that impaired heart function occurs in mice with targeted disruption of ACE2 [66], as values of LV fractional shortening (FS) and velocity of circumferential fiber shortening were severely decreased when compared to wild-type mice. Furthermore, selective blockade of B2 receptors by Hoe 140 reduces coronary blood flow and contractility, and increases left ventricular end- diastolic pressure [67]. We previously mentioned that LV FS was diminished in the infarcted heart, and that FS augmentation was observed after LLLT in infarcted rats. The reduction in infarction size after LLLT was previously described [28], [29]. Herein, using the same energy density from these previous studies, we provide more evidences that LLLT may result in a significant reduction of infarct size.

One of the suggested actions of LLLT is promotion of angiogenesis. However, the laser irradiation parameters used in our MI-LLLT model were not able to enhance VEGF expression or capillaries density. Recent publications have shown an increased angiogenesis after LLLT in several tissues [68]–[71]. This discrepancy between these studies and ours may be explained by the fact that these investigations used different wavelengths and/or energy density in their laser protocols. In fact, changing the energy density may induce different biological actions by LLLT [72]. Thus, further studies must be carried out to achieve a proper dose to increase angiogenesis in infarcted/irradiated myocardium post-MI.

Considering the data presented herein and the current state of knowledge regarding the anti-inflammatory efficacy of LLLT, we conclude that LLLT may exert beneficial effects to the myocardium after MI. The reduction of the infarcted area and attenuation of systolic dysfunction, together with the mRNA upregulation of the protective kinin B2 receptor, the reduction of the kinin B1 receptor and pro-inflammatory interleukins mRNA expressions are in consonance with the expected anti-inflammatory response of LLLT. Increased circulating levels of plasma kallikrein after LLLT reinforces the participation of KKS in the laser protective action in the myocardium. Moreover, the increased expression of ACE2 mRNA and Mas receptor mRNA and protein may suggest the participation of cardioprotective Ang1–7 in the post-MI milieu after LLLT. In addition, eNOS mRNA and NOx concentration upregulation are additional evidences that NO generation, maybe due possible kinin B2 or Mas receptors activation, might be involved in the suggested cardioprotection elicited by LLLT.

Collectively, these findings broaden our understanding of the cardioprotective effect of LLLT on the cardiac tissue, and the relevance of vasoactive systems in relation to the pathophysiology of MI.

### Clinical perspectives and limitations

There have been a considerable number of clinical studies of the responses to LLLT in a broad number of conditions. LLLT applications are safe and usually require only a few minutes to perform. Established protocols and irradiation dosages have been developed that make clinical application relatively simple. Notwithstanding, our study uses an open-chest irradiation model, what makes it impracticable to treat MI in a hospital emergency room. However, laser tissue penetration can be enhanced by changing energy doses and exposition time, leading to photon penetration into the deeper tissues. Our ongoing experiments test different laser irradiation parameters and tissue penetrance in order to achieve better protocols, such as transthoracic irradiation, that might aid MI patients, hopefully, in the near future. Also, a high-efficiency peptides measurement and transgenic models might draw a more accurate analysis of Renin-Angiotensin and Kallikrein-Kinin systems participation in the LLLT cardioprotective action.
